# Identification of Regions Involved in Substrate Binding and Dimer Stabilization within the Central Domains of Yeast Hsp40 Sis1

**DOI:** 10.1371/journal.pone.0050927

**Published:** 2012-12-05

**Authors:** Júlio C. Borges, Thiago V. Seraphim, David Z. Mokry, Fabio C. L. Almeida, Douglas M. Cyr, Carlos H. I. Ramos

**Affiliations:** 1 Institute of Chemistry of São Carlos, University of São Paulo, São Carlos, São Paulo, Brazil; 2 Institute of Chemistry, University of Campinas UNICAMP, Campinas, São Paulo, Brazil; 3 Institute of Biology, University of Campinas UNICAMP, Campinas, São Paulo, Brazil; 4 Institute of Medical Biochemistry, National Center of Nuclear Magnetic Resonance of Macromolecules UFRJ, and National Institute of Science and Technology for Structural Biology and Bioimaging (INBEB), Rio de Janeiro, Rio de Janeiro, Brazil; 5 Department of Cell and Developmental Biology, University of North Carolina, Chapel Hill, North Carolina, United States of America; Semmelweis University, Hungary

## Abstract

Protein folding, refolding and degradation are essential for cellular life and are regulated by protein homeostatic processes such those that involve the molecular chaperone DnaK/Hsp70 and its co-chaperone DnaJ. Hsp70 action is initiated when proteins from the DnaJ family bind an unfolded protein for delivery purposes. In eukaryotes, the DnaJ family can be divided into two main groups, Type I and Type II, represented by yeast cytosolic Ydj1 and Sis1, respectively. Although sharing some unique features both members of the DnaJ family, Ydj1 and Sis1 are structurally and functionally distinct as deemed by previous studies, including the observation that their central domains carry the structural and functional information even in switched chimeras. In this study, we combined several biophysical tools for evaluating the stability of Sis1 and mutants that had the central domains (named Gly/Met rich domain and C-terminal Domain I) deleted or switched to those of Ydj1 to gain insight into the role of these regions in the structure and function of Sis1. The mutants retained some functions similar to full length wild-type Sis1, however they were defective in others. We found that: 1) Sis1 unfolds in at least two steps as follows: folded dimer to partially folded monomer and then to an unfolded monomer. 2) The Gly/Met rich domain had intrinsically disordered characteristics and its deletion had no effect on the conformational stability of the protein. 3) The deletion of the C-terminal Domain I perturbed the stability of the dimer. 4) Exchanging the central domains perturbed the conformational stability of the protein. Altogether, our results suggest the existence of two similar subdomains in the C-terminal domain of DnaJ that could be important for stabilizing each other in order to maintain a folded substrate-binding site as well as the dimeric state of the protein.

## Introduction

DnaJ proteins are ubiquitous co-chaperones of the DnaK/Hsp70 molecular chaperone family, which are involved in the folding of client proteins and in the prevention of aggregate formation [1]–[6]. Although DnaJs display a high degree of diversity in terms of their architectural domains and subcellular localizations, all are characterized by the presence of a J-domain, which is responsible for binding to DnaK/Hsp70 and stimulating its ATPase activity [5], [7]–[10]. In addition, DnaJs bind to client proteins through hydrophobic interactions and present them to DnaK/Hsp70 [11]–[13], but some can also function as chaperones through their own activity [11], [14].

**Figure 1:**
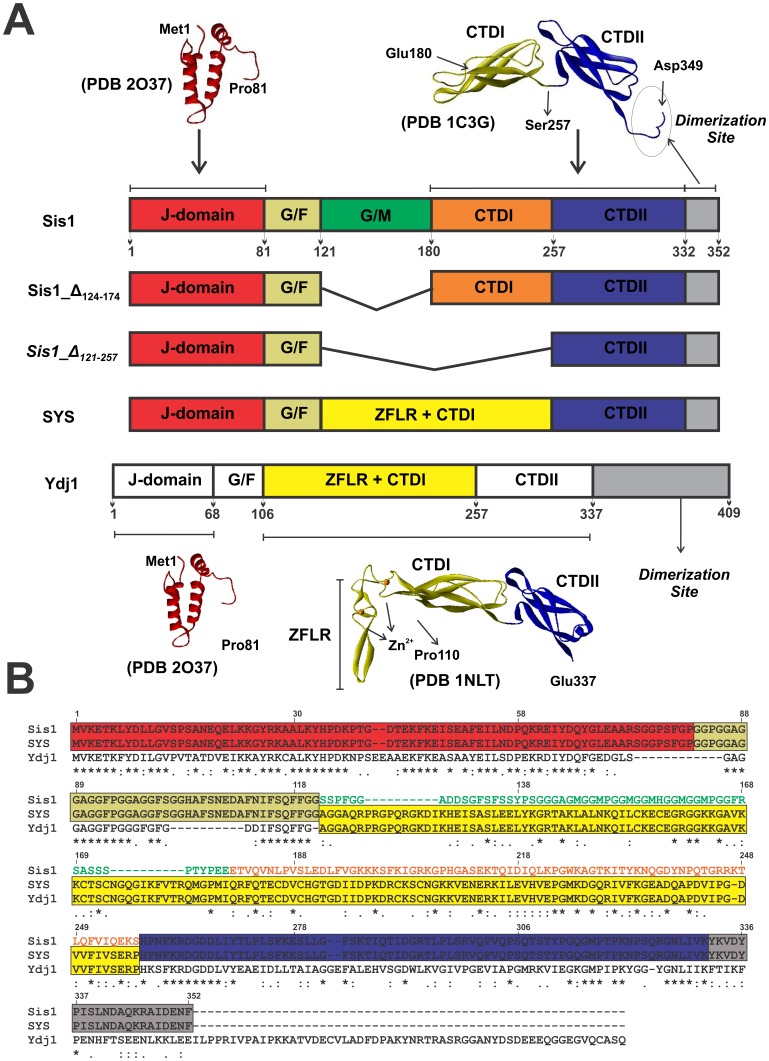
Structural domain arrangements of wild-type Sis1 and mutants. (**A**) Sis1 contains the J-domain and the G/F region located in the N-terminal region (121 amino acids residues – red box). In the central portion, between the G/F domain and the C-terminus, Sis1 has a G/M-rich (Glycine/Methionine-rich – green box) domain and Ydj1 has a zinc-finger-like region (ZFLR; yellow box). The C-terminal domains (CTD) I and II follow (orange and blue boxes, respectively). In Sis1_Δ_124–174_ the G/M region is absent and in Sis1_Δ_121–257_ both the G/M region and the CTDII are deleted. The chimera SYS is formed by the J-domain and the CTDII from Sis1 and the middle region from Ydj1. Ydj1 is shown for comparison. PDB accession numbers: 2O37 (red, residues 1–81), 1C3G (yellow, residues 180–255; blue: 256–349) [16], 1NLT (yellow, residues 110–255 and blue 256–337) [13]. (**B**) Amino acid sequence alignment of Sis1, SYS and Ydj1. Red box, J-domain; Green, G/M-rich region; red, CTDI; blue, CDTII; yellow, permuted region between Ydj1 and Sis1; gray, dimerization region of Sis1.

DnaJ proteins contain four conserved domains/regions (Fig. 1) [3], [7]. The highly conserved α-helical J-domain defines DnaJ proteins and is at the N-terminus in Types I and II. Secondly, a disordered Gly/Phe rich region (G/F region) is present which is responsible for flexibility and may function as a linker between the J-domain and the C-terminal region of DnaJ proteins [15]. Beyond that, there is no conservation among Type I and II, although some Type II proteins share some C-terminal homology with Type I proteins, but this is not a necessary criteria.

The sequence and position of the domains defines DnaJ Types I and II [3], [7]. In Type I, such as in yeast Ydj1, the central portion between the G/F domain and the C-terminus contains a zinc-finger-like region (ZFLR) whereas in Type II, such as in yeast Sis1, this is replaced by a G/M-rich (Glycine/Methionine-rich) domain (Fig. 1) [1], [8]. The region known as the C-terminal Domain (CTD) is formed by two structurally similar subdomains (named CTDI and CTDII) composed of β-sheet structure (see Fig. 1A). These two domains dimerize through the C-terminus region and fold into a bent horseshoe dimer [13], [16]. Both the CTD and ZFLR are responsible for the chaperone activity of Hsp40s [11]–[13].

Interestingly, Type I and II DnaJs are not equivalent. For example, Ydj1 and Sis1 display divergent activities on yeast Hsp70 and chaperone function [17], [18]. Fan et al. [19] present interaction data among chaperone substrate models for Ydj1 and Sis1 and two engineered chimeras in which the central part of Sis1 (residues 122–257) was exchanged with the central part of Ydj1 (residues 101–255) and vice versa, generating the chimeras SYS and YSY, respectively. These chimeras switch the specificity for binding substrates and in stimulating firefly luciferase refolding activity by DnaK/Hsp70 [19]. These results suggest that DnaJ substrate-binding sites are needed for substrate selection in DnaJs, and that the central portion of this protein is important for chaperone activity specification [19]. In addition, human Type I and II DnaJs have divergent structures and substrate binding selectivity, possibly regulating the activity of DnaK/Hsp70 by multiple binding site interactions [20]. Similarly, structural solution studies suggest that the switch of the central part of Sis1 and Ydj1 also induced an exchange in the structures of the chimeras (SYS and YSY) [21]. Interestingly, deletion of the central part of Sis1 did not result in a large change in its overall shape, whereas deletion of the central part of Ydj1 largely altered its overall shape to a conformation similar to that seen for Ydj1 [22]. All of these observations directly suggest that the central region of DnaJs encodes their structure.

**Figure 2:**
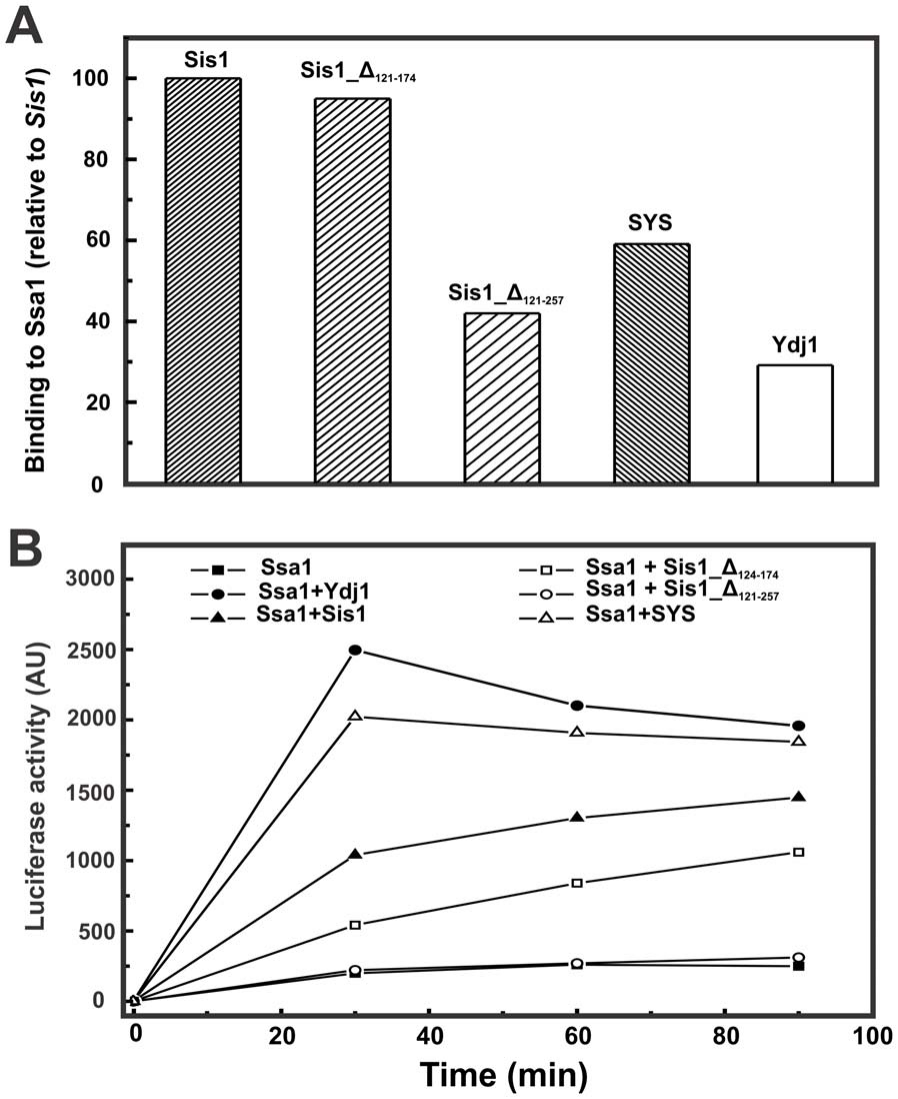
Functional tests of wild-type Sis1 and mutants and Ydj1. The ability of yeast DnaJ and mutants in binding to Hsp70 Ssa1 (**A**) and in cooperating with Hsp70/Ssa1 to refold heat-denatured firefly luciferase (**B**) was monitored. Sis1 activity was set as a standard (100%).

In this study, we applied unfolding strategies for studying the stability of Sis1, two deletion mutants of its central region (Sis1_Δ_124–174_ and Sis1_Δ_121–257_) and a chimera formed by the central region of Ydj1 and the N- and C-terminal regions of Sis1 named SYS (Sis1_1–121_-Ydj1_101–255_-Sis1_258–352_) (Fig. 1) [19]. Based on thermal unfolding followed by differential scanning calorimetry (DSC), chemical unfolding followed by circular dichroism (CD), analytical ultracentrifugation (AUC), and size exclusion chromatography coupled to multi-angle laser light scattering (SEC-MALLS) studies, we suggest that Sis1 unfolds in at least two steps as follows: folded dimer to partially folded monomer and then to an unfolded monomer. The importance of the C-terminus to Sis1 stabilization and dimerization is addressed below. In addition, we observed by thermodynamic data and nuclear magnetic resonance (NMR) experiments that the G/M region of Sis1 is intrinsically disordered.

**Figure 3:**
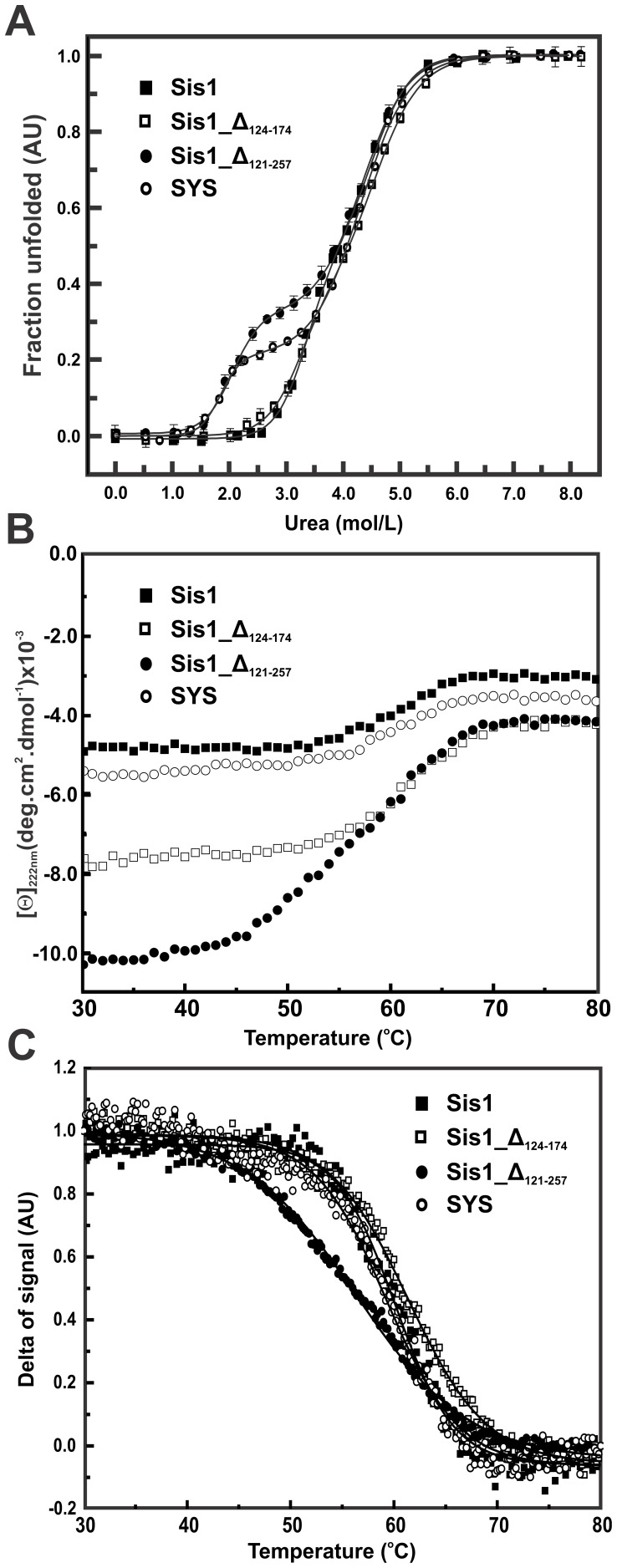
Urea- and thermal-induced unfolding measurements followed by CD. The recombinant proteins (0.5 mg/mL) were submitted to thermal-unfolding followed by CD at 222 nm. (**A**) Urea-induced unfolding experiments were followed by CD at 222 nm, using a 1 mm pathlength cell in buffer A, at 20°C after 90–120 min of equilibration. Data are shown as fraction of unfolded protein and represent the mean of three independent experiments. The three-state unfolding transition model (Eq. 1) was used to fit the fraction of unfolding data and the fitting is shown by the line (see text for details). The unfolding experiments (in triplicate) were measured from 20°C to 90°C with a scan rate of 1.0°C/min. Data is presented as [θ] (**B**) and in delta of signal (**C**), which was fitted using a sigmoidal function (full line) yielding the *Tm_CD_*.

## Materials and Methods

### Protein Preparation and Chaperone Activity

All recombinant proteins were expressed by *Escherichia coli* BL21(DE3) cells and purified by two chromatography steps as previously described, unless otherwise noted [19], [21], [22]. For the biophysical experiments performed in this study, all proteins were prepared in buffer A (25 mM Tris-HCl, pH 7.5, 500 mM NaCl). Protein concentrations were determined by the Edelhoch method. The activities of Sis1 and mutant variants were tested by their ability to cooperate with the Hsp70 Ssa1 to refold heated denatured firefly luciferase [12], [19]. The ability of Sis1, mutants and Ydj1 in binding to Hsp70 Ssa1 was tested by a modified enzyme-linked immunosorbent assay (ELISA) [12], [19]. Sis1 and variants (100 µL at 200 nM), in 50 mM phosphate (pH 7.4), 150 mM NaCl, were loaded into a microplate and after incubation for 1 h the wells were washed to remove the unbound proteins with 50 mM phosphate (pH 7.4), 150 mM NaCl, and 0.02% Triton X-100 (washing solution). Then, the wells were blocked by 1 h incubation with 200 µL of 0.5% of bovine serum albumin in 50 mM phosphate (pH 7.4), 150 mM NaCl, and washed again. Ssa1 (100 µL at 100 nM) prepared in 50 mM phosphate (pH 7.4), 150 mM NaCl and 5 mM Mg-ATP, was incubated for 1 h after which the wells were washed with washing solution, followed by incubation for 1 hour with anti-rabbit α-Ssa1. The wells were washed five more times with the washing solution, and then the goat anti-rabbit horseradish peroxidase secondary antibody was applied and incubated for 45 minutes. After three washes, the peroxidase substrate solution was added to the wells to detect the bound anti-rabbit α-Ssa1, and the color formation was measured using a microplate reader at 415 nm. All reactions were done in the presence of 5 mM Mg-ATP at 25°C. Elisa assays were run to confirm that equal quantities of proteins were retained in each well of the plates.

**Figure 4:**
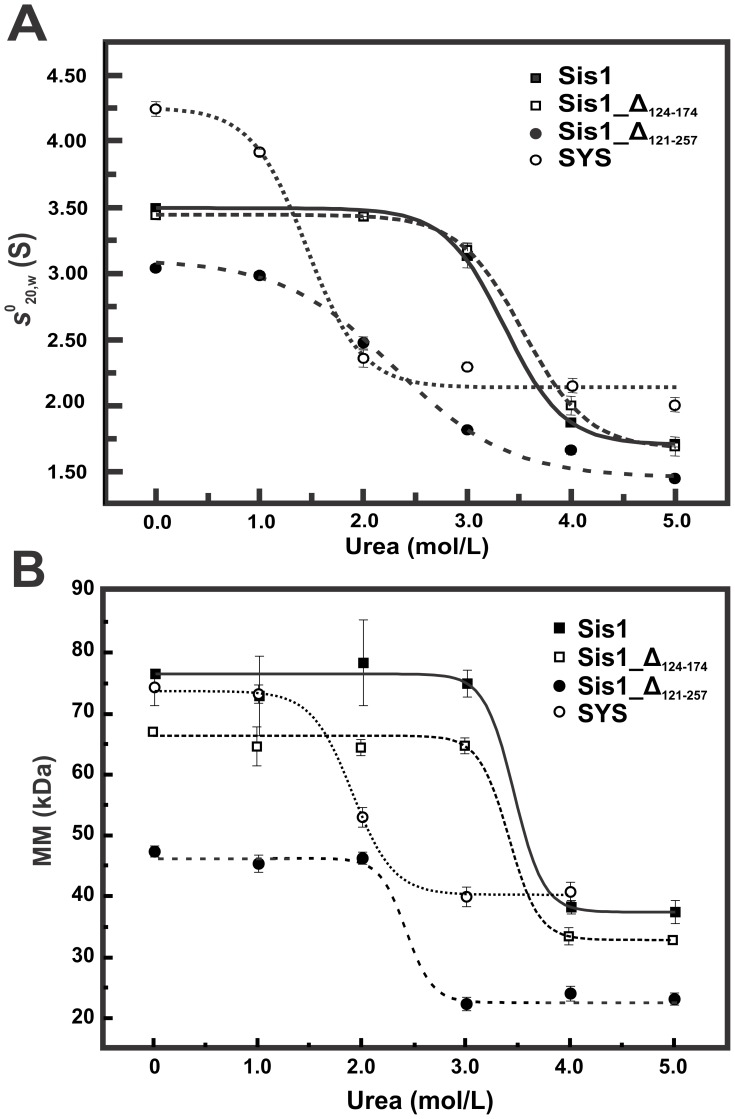
Urea disturbs the dimer structure of Sis1_Δ_121–257_ and SYS at lower concentrations than for Sis1 and Sis1_Δ_122–174_. (**A**) SV-AUC experiments were done for all proteins (250–750 µg/mL) in the presence of urea at concentrations ranging from 1–5 M in buffer A. The curves of *s* ^0^_20,w_ versus urea concentrations were fitted by sigmoidal functions (continuous line) leading us to obtain the *Cm_AUC_* (Table 1). Data at 0 M urea were obtained from [22] and [21]. (**B**) SEC-MALLS experiments were performed with all proteins in concentrations from 50 µM to 75 µM and in the presence of urea at concentrations ranging from 1–5 M in buffer A. The curves of MM *versus* urea concentration were fitted by sigmoidal functions (lines) resulting in the *Cm_SEC-MALLS_*, which represents the urea concentration of the midpoint of the MM transition (Table 1). The molecular masses for wild-type Sis1, SYS, Sis1_Δ_124–174_ and Sis1_Δ_121–257_ were approximately 78, 75, 68 and 48 kDa in the absence of denaturant, and approximately 39, 42, 35 and 24 kDa in 4 M urea, respectively. Taken together, the results suggest that Sis1 and Sis1_Δ_124–174_ are dimers at higher urea concentrations than SYS and Sis1_Δ_121–257_.

### Circular Dichroism

Circular Dichroism (CD) measurements were performed in a Jasco J-810 spectropolarimeter coupled to a Peltier-type temperature control *System* PFD 425S. The proteins were tested in buffer A at final concentrations from 5 µM to 20 µM. Chemical-induced unfolding followed by CD was performed using a urea concentration gradient ranging from 0–6 M, after a 90 min equilibration at room temperature (∼20°C). The CD signal at 222 nm was acquired using a 1 mm pathlength cuvette, at 20°C, for each fresh urea solution prepared in buffer A. The chemical reversibility was tested by diluting proteins from a 6 M urea solution to a 1 M urea solution followed by collection of the CD spectra. All experiments were done in triplicate and all reagents were of chemical grade. The chemical-induced unfolding profiles were transformed to fraction of unfolded protein and fitted using the following equation considering a three-state unfolding model.
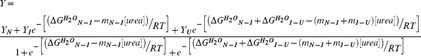
(1)


**Table 1:** Summary of the thermodynamic data obtained from unfolding experiments.

Thermodynamic Property	Protein			
	Sis1	Sis1_Δ_124–174_	Sis1_Δ_121–257_	SYS
*ΔG* ^H^_2_^O^ *_N-I_* (kcal/mol)	7.2±0.5	6.2±0.2	4.8±0.6	6.0±0.8
*ΔG* ^H^_2_^O^ *_I-U_* (kcal/mol)	8.0±0.1	6.6±0.8	6.3±0.3	5.9±0.2
*m_N-I_ (kcal/mol.M)*	2.1±0.2	2.0±0.2	2.4±0.3	3.3±0.4
*m_I-U_ (kcal/mol.M)*	1.7±0.2	1.5±0.2	1.5±0.6	1.4±0.4
*Cm_N-I_* (M)†	3.3±0.1	3.2±0.1	2.0±0.1	1.8±0.1
*Cm_I-U_* (M)†	4.5±0.1	4.5±0.2	4.3±0.1	4.3±0.1
*Cm_AUC_* (M)‡	3.4±0.1	3.5±0.1	2.1±0.2	1.4±0.1
*Cm_SEC-MALLS_* (M)‡	3.5±0.1	3.4±0.1	2.4±0.2	1.9±0.1
*Tm_1_* (°C)	59.0±0.2	59.2±0.4	53.8±0.2	45.1±0.7
*Tm_2_* (°C)	67.7±0.6	67.3±0.2	65.6±0.5	63.7±0.4
*ΔH_1_^cal^* (kcal/mol)	154.0±0.2	149.3±0.5	70.3±0.5	84.3±0.8
*ΔH_2_^cal^* (kcal/mol)	29.5±0.5	29.3±0.2	48.2±0.6	59±0.1
*ΔCp_1_* (kcal/mol/K)*	4.0±0.4	5.1±0.6	3.1±0.4	3.8±0.1
*ΔCp_2_* (kcal/mol/K)*	1.3±0.4	1.4±0.2	1.4±0.5	1.7±0.7
*Tm_CD_* (°C)	60.1±0.5	61.4±0.5	56.4±0.5	59.3±0.5

* Data estimated from the Kirchoff plot by application of Eq. 3 (Fig. 3).

† determined from the *ΔG^w^_N-I_*/*m_N-I_ and ΔG^w^_I-U_*/*m_I-U_* ratio;

**Figure 5:**
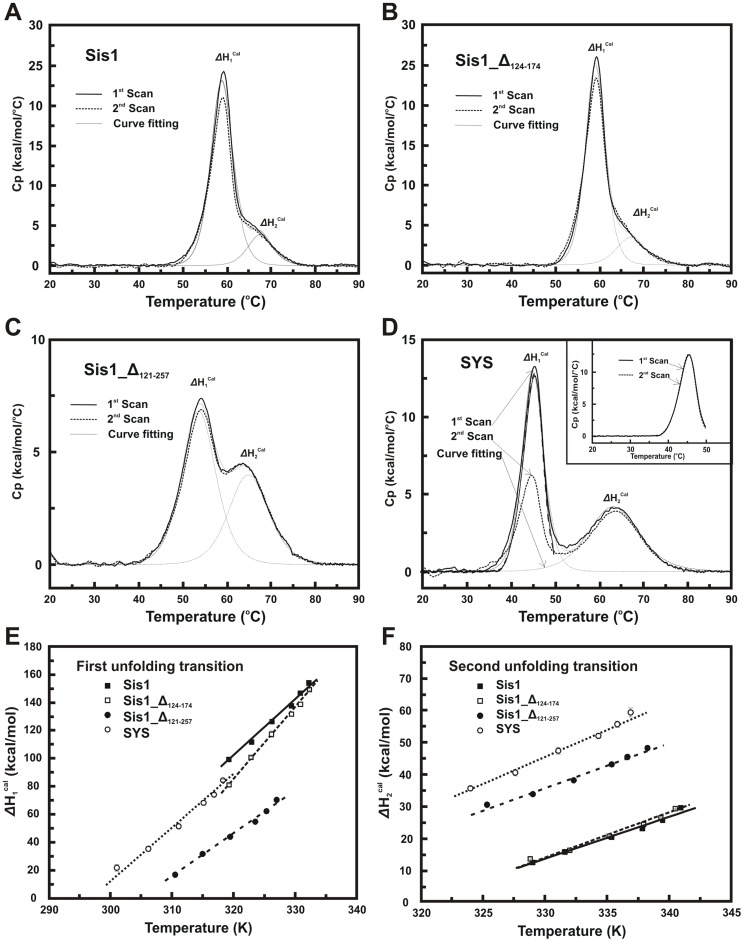
Thermal-induced unfolding measurements followed by DSC. (**A**) Sis1; (**B**) Sis1_Δ_124–174_; (**C**) Sis1_Δ_121–257_ and (**D**) SYS. The figures represent the first and second scans after baseline treatment. Sis1 and Sis1_Δ_124–174_ showed two overlapping transitions with similar *Tm*s of approximately 59 and 67°C (Table 1). Sis1_Δ_121–257_ and SYS showed two well separated unfolding transitions where the second Tm was within 64–66°C (Table 1). For all proteins, the second unfolding transition was more than 95% reversible. The first unfolding transition of Sis1, Sis1_Δ_124–174_ and Sis1_Δ_121–257_ was approximately 90% reversible when the proteins were heated to 90°C. SYS (Fig. D) presented a reversibility of approximately 60% when heated to 90°C. However, when SYS was heated to 50°C (Fig. **D**, inset) it was more than 95% reversible. Similar behaviors were observed with other proteins. **E–F)** Kirchoff plots showing the dependence of *ΔH* ^cal^ with *Tm* for *Sis1* and its mutants. Urea concentrations from 0.25 M to 2.0 M were used in order to disturb both *ΔH* ^Cal^ and *Tm* that were monitored by DSC. The figure shows the Kirchoff plot for the first (**E**) and second (**F**) thermal-induced unfolding transition. The curves were fitted using Eq. 3 in order to obtain the *ΔCp* for each unfolding transition.

‡ *Cm_AUC_* and *Cm_SEC-MALLS_* are the urea concentrations of the midpoint of the *s* ^0^_20,w_ and MM transition, respectively (Fig. 5).

Y_N_, Y_I_ and Y_U_ are the signals for fraction of unfolded protein for native, intermediate and unfolded species, respectively. *R* and *T* are the gas constant and absolute temperature, respectively. Subscripts N-I and I-U are the native-intermediate and intermediate-unfolded transitions, respectively. *ΔG^H^_2_^O^_N-I_* and *ΔG^H^_2_^O^_I-U_* are the free energy change in the absence of denaturant, and m_N-I_ and m_I-U_ are the m-values for each transition. *Cm_N-I_* and *Cm_I-U_* are the urea concentrations at the midpoint of the unfolding transitions, which were determined by the *ΔG^H^_2_^O^_N-I_/m_N-I_* and the *ΔG^H^_2_^O^_I-U_/m_I-U_* ratios, respectively. In the case of the proteins studied here, native means the folded dimer and unfolded means the unfolded monomer. The intermediate means an unknown species between the two aforementioned species. Thermal-induced unfolding experiments followed by CD were performed at a temperature rate of 1°C/min using a 1 mm pathlength cell. The average of three unfolding curves was used to build the thermal-unfolding profile which was transformed to fraction of unfolded protein curves. The temperature of the midpoint transitions (*Tm*) were estimated by fitting the thermal-unfolding profile with sigmoidal functions.

### Hydrodynamic Characterization

The effect of urea on the oligomeric structure of the proteins was tested using Analytical Ultracentrifugation (AUC) and Size Exclusion Chromatography - Multi-Angle Laser Light Scattering (SEC-MALLS). Sedimentation velocity (SV) AUC experiments were performed in a Beckman Optima XL-A analytical ultracentrifuge. The protein concentration used ranged from 250–750 µg/mL, and the rotor speed was 40,000 rpm (20°C). The samples were prepared in different urea concentrations (1.0–5.0 M) from a fresh urea stock solution prepared in buffer A. The SedFit software (Version 9.4) was applied in order to fit the absorbance versus cell radius data. The SedFit program deconvolutes sedimentation and diffusion to solve the Lamm equation [23] in order to obtain both the continuous sedimentation distribution *c(S)* and a weight average value of frictional ratio (*ƒ/ƒ*_0_) which was the parameter of function regularization. The apparent sedimentation coefficients (*s*) were obtained as the maximum of the peak of the *c(S)* curve. The experimental *s*-values contain interferences caused by viscosity and density increments at different urea concentrations, therefore we estimated the standard sedimentation coefficients (*s*_20,w_) at each protein concentration from the apparent *s* [24]. The Sednterp software (www.jphilo.mailway.com/download.htm) was used in order to estimate the urea contribution to buffer viscosity and density and also to correct the apparent *s* to *s*_20,w_ at all SV experiments performed in the presence of urea. We calculated, by linear regression, the standard sedimentation coefficient at 0 mg/mL protein concentration (*s* ^0^_20,w_) from values of *s*_20,w_ versus protein concentrations. The Sednterp software was applied to estimate the partial specific volume (*V_bar_*) for Sis1 (0.7263 mL/g), Sis1_Δ_124–174_ (0.7312 mL/g), Sis1_Δ_121–257_ (0.7284 mL/g) and SYS (0.7296 mL/g) from their amino acid sequences.

**Figure 6:**
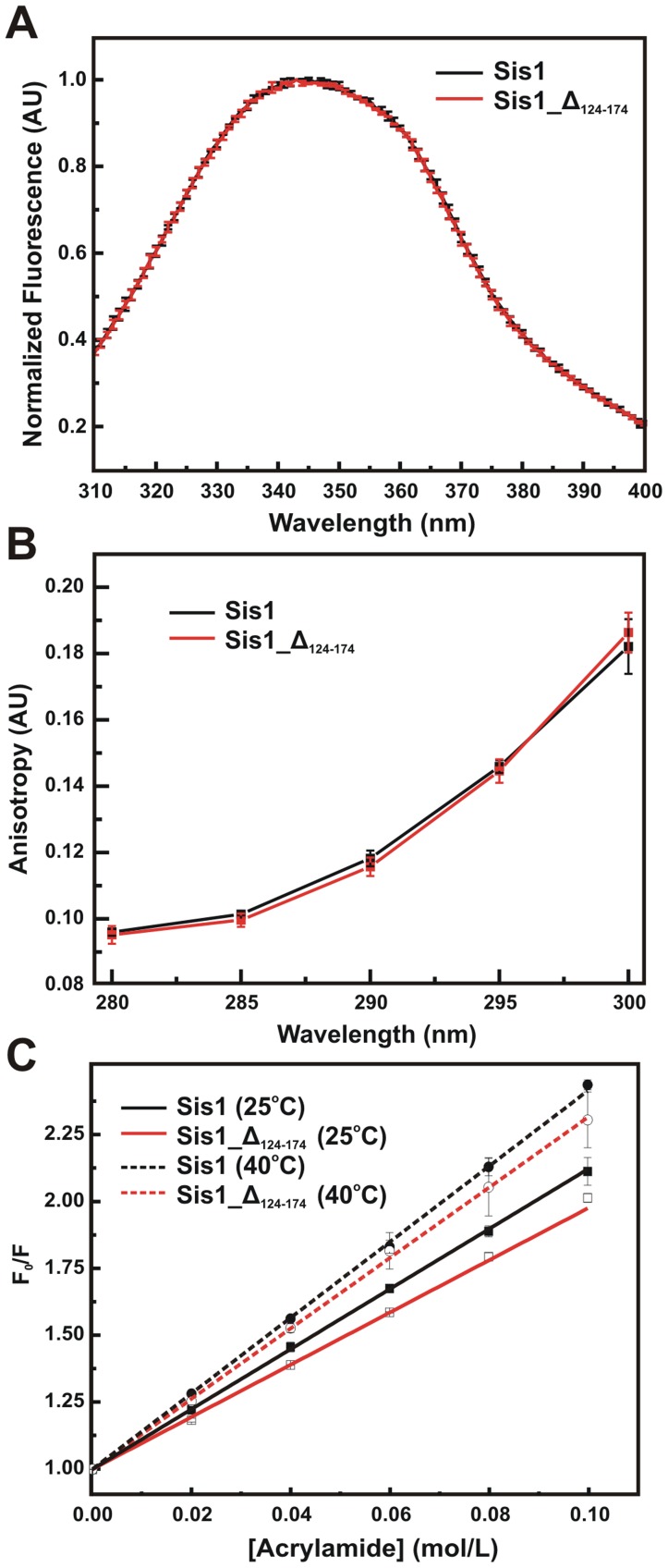
Fluorescence studies of Sis1 and Sis1_Δ_124–174_. The lone tryptophan residue in Sis1 and Sis1_Δ_124–174_ (5 µM protein concentration) was used as a probe to investigate changes in the local protein structure and in hydrodynamic parameters, such as rotational diffusion, caused by the deletion of 50 amino acids in Sis1. The (**A**) fluorescence emission spectra, (**B**) tryptophan anisotropy and (**C**) tryptophan quenching results are shown. All experiments were performed in triplicate.

The molecular masses (MM) of the proteins were also analyzed by SEC-MALLS using a Tricorn Superdex 200 10/300 GL (GE Healthcare) coupled to a miniDAWN TREOS device (Wyatt Technologies), both connected to an ÄKTA FPLC (GE Healthcare). The SEC-MALLS experiments were performed at a flow rate of 0.5 mL/min after column equilibration with 1 column volume and the light scattering at 90° less than 0.02 V. The light scattering and absorbance at 280 nm were treated with the program ASTRA (Wyatt Technologies). Experiments in the absence of urea were done using a protein concentration of 25 µM prepared in buffer A. SEC-MALLS experiments with proteins (50–75 µM) in the presence of urea from 1 to 5 M prepared in buffer A were incubated for 1 h at room temperature.

**Table 2:** Summary of fluorescence experiment data. Errors are shown inside parentheses.

Experimental data		Protein	
		Sis1	Sis1_Δ_124–174_
Maximum λ of fluorescence (nm)		343	343
Center of spectral mass (nm)		343	343
Stern-Volmer constant (M^−1^)	25°C	11.1±0.1	9.7±0.1
	40°C	14.1±0.1	13.1±0.1

### Differential Scanning Calorimetry

The measurements of thermal unfolding were done in a VP-DSC Differential Scanning Calorimeter (MicroCal), at a protein concentration of 500 µg/mL prepared in buffer A after extensive dialysis. The scan rate tested was 1.0°C/min at a temperature range of 15–90°C. The reversibility of thermal unfolding was tested by performing several consecutive up/down scans and reversibility was accepted if the area under the curve of each heat capacity transition was maintained in the consecutive scans greater than 95%.

**Figure 7:**
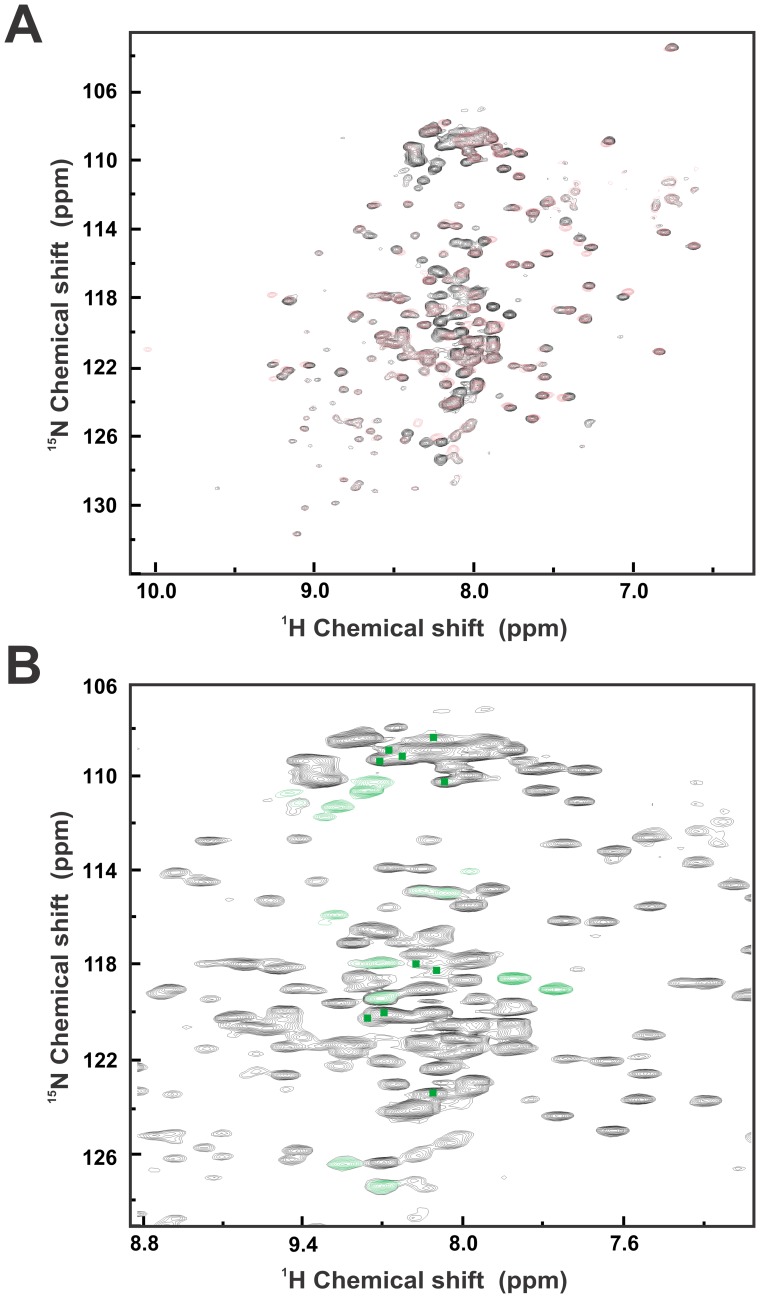
NMR experiments for Sis1 and Sis1_Δ_124–174_. (**A**) Superimposed TROSY NMR spectra of Sis1 (black signals) and Sis1_Δ_124–174_ (red signals) showing that Sis1_Δ_124–174_ presents less signals, all of them in the central region of the TROSY spectrum with chemical shifts around 8.2. (**B**) Zoom of the 2D ^1^H ^15^N chemical shifts of Sis1 showing the crosspeaks (marked in green) which are present in the wild-type Sis1 spectra but absent in the Sis1_Δ_124–174_ spectra. The NMR experiments suggest that the deleted region of Sis1 is intrinsically disordered.

The thermograms were collected using a Microcal VP-Viewer 2000 program and fitted using the Microcal Origin 5.0 program, both supplied with the device. The fitting routine used a non-two state unfolding model. The baselines were calculated from the pre- and post-transition temperature regions. The apparent calorimetric enthalpy change of the unfolding transition (*ΔH* ^cal^) was calculated by integrating the area under the peak of the heat capacity (Cp^trs^) of the unfolding transition using the following equation:
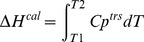
(2)where *T* is the temperature. *Tm* was estimated as the midpoint of transition.

**Figure 8:**
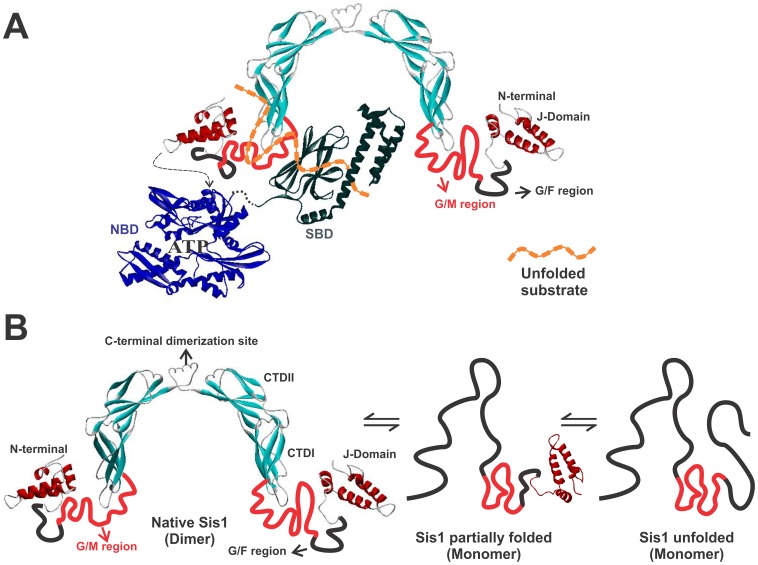
A) Model of the Sis1 binding and delivery. A model that combines Sis1 observations showing that the disordered G/M region participates in the binding and delivery of client proteins to Hsp70. A schematic Sis1 structure is represented as a dimer showing its main domains and regions. Light blue, Sis1 CTD (PDB accession number 1C3G); red, Sis1 J-domain (PDB accession number 2O37); dark blue, Hsp70 NBD (PDB accession number 1HJO); green, DnaK SBD (PDB accession number 1DKX). The unstructured G/F and G/M regions of Sis1 are presented in black and red lines, respectively. The schematic substrate is in orange. **B) Model of the Sis1 unfolding pathway.** We propose a model for the Sis1 unfolding pathway which involves at least 2 events based on the results obtained in this study (see text for details).

Only the first scan was used for analysis since some proteins displayed a partially reversible first transition if heated to 90°C, although reversibility was confirmed by heating to the *Tm* (see below). Thus, for statistical purposes, we carried out three independent thermal unfolding experiments at each condition in order to obtain a set of first-scan thermograms. Considering the inherent errors in the determination of the baseline during the first scan of excess calorific capacity, and the complexity of the thermograms (see below), we estimated the heat capacity change (*ΔCp*) for each unfolding transition from the Kirchoff plot (Eq. 3). This plot represents the dependence of the *ΔH* ^cal^ (the subscripted number aforementioned indicates the related unfolding transition) with the *Tm*.
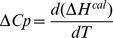
(3)


**Table 3:** Estimation of *ΔCp_residue_* (the ratio of the *ΔCp* per amino acid residue of the predicted domain) values for the first and second transitions observed in DSC experiments.

Protein	CTD		J-domain+G/F region (121 residues)
	number of amino acids	*ΔCp1_residue_* (cal/mol/K)	*ΔCp2_residue_* (cal/mol/K)
*Sis1*	235	17.0±0.2§	11.0±0.1¶
*Sis1_Δ_124–174_*	180	28.5±0.3§	11.8±0.1¶
*Sis1_Δ_121–257_*	96	32.0±0.4§	11.8±0.2¶
*SYS*	252	15.1±0.3§	14.0±0.5¶

§ from the ratio of *ΔCp_1_* by the number of amino acids present in the CTD of each protein*;*

¶ from the ratio of *ΔCp_2_* by 121 residues which corresponds to the length of the J-domain plus the G/F region of *Sis1;*

To disturb both *ΔH* ^Cal^ and *Tm* we used urea as a denaturing agent. The experiments were done in the absence or presence of urea (0.25–2 M) prepared from a fresh solution in buffer A.

**Figure 9:**
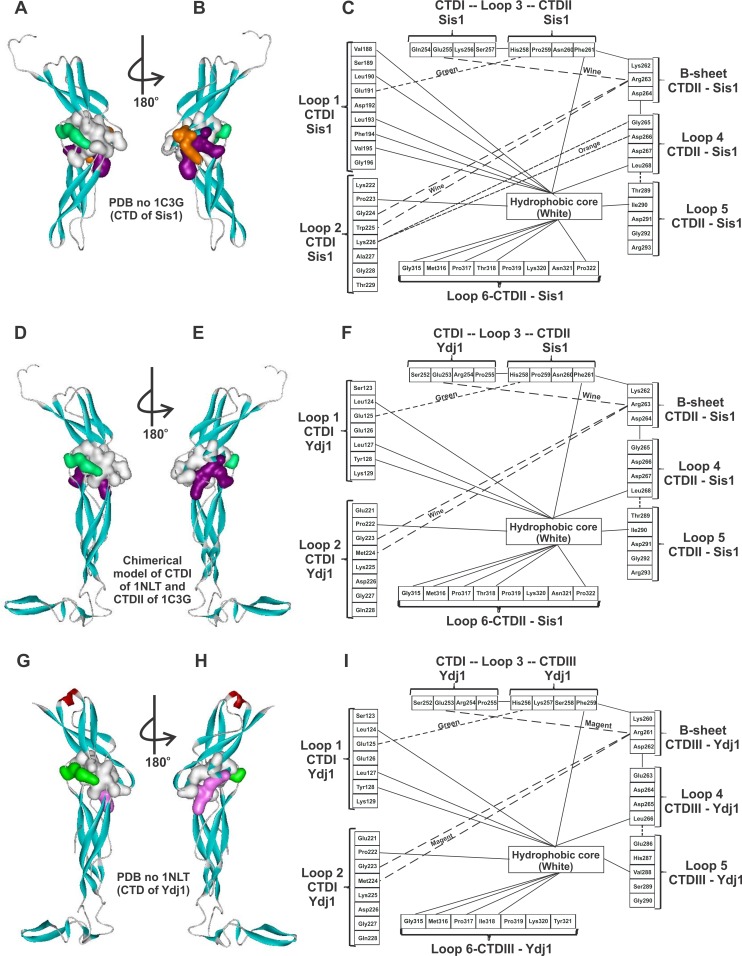
Interface contacts between CTDI and CTDII of Sis1, SYS and Ydj1. The contacts between CTDI and CTDII of the crystallographic model of Sis1 (**A** and **B**) and Ydj1 (**G** and **H**) were scouted and compared to the chimerical model of the CTD of SYS (**D** and **E**) generated by molecular homology modeling (data not shown). Panels **C**), **F**) and **I**) present the amino acids at the interface that make hydrophobic contacts (full line) and electrostatic contacts (dashed lines). Some of the later contacts involve atoms of the peptide bond of the indicated amino acids. Amino acids not indicated are on the surface or turned inwards towards the core of the CTD subdomains.

### Fluorescence Experiments

Fluorescence experiments were performed in a SLM2 Aminco Bowman spectrofluorimeter with a protein concentration of 5 µM in buffer A. The center of spectral mass (<λ>) was calculated using the equation below:
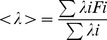
(4)where λi is the fluorescence emission wavelength and *Fi* is the fluorescence intensity measured. For quenching experiments, the proteins were incubated with increasing acrylamide concentrations ranging from 0 to 100 mM and the data were recorded at 25°C and 40°C. The Stern-Volmer constants for wild-type Sis1 and Sis1_Δ_124–174_ were calculated using the Stern-Volmer equation for collisional quenching processes:
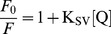
(5)where, *F* and *F*_0_ are the tryptophan emission fluorescence at the maximum intensity wavelength with and without the quencher agent, respectively; *K*_SV_ is the Stern-Volmer constant; and [*Q*] is the concentration of the quencher agent, the acrylamide. The fluorescence anisotropy measurements were done in an ISS K2 spectrofluorometer coupled with excitation and emission polarizers. The tryptophans in 5 µM of protein in buffer A were excited at wavelengths from 280 to 300 nm and the fluorescence intensities at 343 nm were recorded. The data were analyzed by VINCI program (ISS), yielding the values of anisotropy. All experiments were performed using a 0.2×1 cm pathlength cell.

**Table 4:** Number of amino acids in the subdomain interface that are in contact.

Structural model	Sis1 - PDB 1C3G		SYS chimera&		Ydj1 - PDB 1NLT	
Domain	CTDI	CTDII	CTDI	CTDII	CTDI	CTDIII
Number of amino acids in hydrophobic contact at thesubdomain interface	6	8	4	8	4	7
Total	14		12		11	
Number of amino acids in electrostatic contacts at thesubdomain interface	5	3	4	2	4	2
Total of electrostatic contacts	6		4		4	

& Chimerical model presented on Fig. 9 (D–F).

### Nuclear Magnetic Resonance Experiments

Wild-type Sis1 and Sis1_Δ_124–174_ were labeled with ^15^N and ^2^H using an existing protocol with slight modifications [25]. In brief, we inoculated 2–3 colonies of *E. coli* BL21(DE3) pLysS transformed with the desired expression plasmid into 5 mL of LB media containing 50 µg/mL ampicillin and 50 µg/mL chloramphenicol and allowed the cells to grow for 3 hrs at 37°C. The cells were then harvested (2,500 g for 15 min) and resuspended in 20 mL normal M9 media (32.5 mM Na_2_HPO_4_, 22 mM KH_2_PO_4_, 8.5 mM NaCl, 18.7 mM NH_4_Cl, 1.0 mM MgSO_4_, 0.6% glucose, 50 µg/mL ampicillin, and 50 µg/mL chloramphenicol) and allowed to grow to an A_600_ ≈ 0.6. Cells were then harvested (2,000 g for 15 min) and resuspended into 50 mL of ^15^N marked M9 media containing ^15^NH_4_Cl in place of ^14^NH_4_Cl, and 99% D_2_O and allowed to grow at 37°C until the A_600_ reached between 0.4 and 0.5, at which time 450 mL of additional ^15^N marked M9 media in 99% D_2_O was added to the cell suspension. The growth of the cells was then monitored until the A_600_ reached between 0.3 and 0.4, at which time induction with 0.5 mM IPTG (solubilized in D_2_O) was performed for 4 hrs at 30°C. The cells were then harvested (2,500 g for 15 min), frozen and stored at −80°C until further use. The proteins were purified by a one step cationic exchange chromatography using a High S resin (BioRad), and the desired fractions were concentrated in a centrifugal concentrator device. The final protein concentration of the samples used in the NMR experiments was 145 µM for the wild type and 121 µM for the mutant in 20 mM HEPES, pH 7.5 and 250 mM NaCl. NMR spectra were acquired in a Bruker Avance III spectrometer operating at 800.13 MHz using a room temperature TXI triple resonance probe. Transverse relaxation optimized spectra (TROSY) [26] were acquired for the ^2^H and ^15^N double labeled Sis1 and Sis1Δ_124–174_ at 298 K, using 1024 complex points in the direct dimension and 200 complex points in the indirect dimension and 32 transients accumulated. We used echo-antiecho-TPPI [27] for coherence selection and quadrature detection in the indirect dimension.

## Results

### The Proteins and their Functions

All proteins studied here contain the J-domain and G/F region, as does native Sis1, located in the N-terminal region (121 amino acids residues). However, they differ in the central portion and in the CTD composition (Fig. 1). All proteins expressed well, and purified in their folded state and behaved as dimers in solution as previously described [21], [22].

Some functional aspects of Sis1 and its mutants and Ydj1 were evaluated (Fig. 2). Wild-type Sis1 and Sis1_Δ_124–174_ showed similar binding ability to Ssa1 by ELISA while mutants Sis1_Δ_121–257_ and SYS and full-length Ydj1 showed roughly 50% and 30% respectively, of the ability observed for Sis1 (Fig. 2A). The ability of the proteins in stimulating Hsp70 *Ssa1* to refold heat-denatured firefly luciferase was also monitored as a function of time and is shown in the Fig. 2B. Full-length Ydj1 and SYS had a stronger effect than wild-type Sis1, while Sis1_Δ_124–174_ had a reduced effect in comparison to wild-type Sis1 and Sis1_Δ_121–257_ showed no effect at all (Fig. 2B). This functional assay suggests that both the G/M region and the CTDI in native Sis1 perform essential roles in substrate selection and/or in its delivery to Hsp70.

### Chemical-induced Unfolding

The stabilities of the proteins were investigated by urea-induced unfolding followed by CD (Fig. 3A), SV-AUC (Fig. 4A) and SEC-MALLS (Fig. 4B). The curve profiles from CD for wild-type Sis1 and Sis1_Δ_124–174_ apparently had one transition while the curve profiles for Sis1_Δ_121–257_ and SYS apparently had two transitions. *ΔG^H^_2_^O^*, *m-value* and *Cm* from the fitted curves using a three state equation and the fitting parameters are presented in Table 1. The *Cm*s for the first transition of wild-type Sis1 and Sis1_Δ_124–174_ are virtually the same inside the error and much higher than those of Sis1_Δ_121–257_ and SYS, which are identical inside the error (Table 1). However, there were no detected differences in the *Cms* of the second transition of the proteins (Table 1). The values of *Cm* were used for comparison due to the uncertainties in calculating ΔG^H^_2_^O^ and the *m-value* with high precision [28], [29].

SV-AUC was used to measure *s* ^0^_20,w_ as a function of urea concentration (Fig. 4A). Wild-type Sis1 and Sis1_Δ_124–174_ were similarly affected by the presence of urea, the *s* ^0^_20,w_ was approximately 3.5 S in 0 to 3 M urea and changed to 2 S in 4 to 5 M urea (Fig. 4A). For Sis1_Δ_121–257_ the transition from approximately 3.1 S to 1.7 S occurred in 2 to 3 M urea, whereas for *SYS* the transition from 4.0 S to approximately 2.7 S occurred in 1 to 3 M urea (Fig. 4A). Similar profiles were identified when the molecular masses of the proteins measured by SEC-MALLS were followed as a function of urea concentration (Fig. 4B). All proteins were dimers in at least 1 M urea and were monomers in 4 M urea (or even at lower urea concentrations depending on the mutation, see Fig. 4B).

### Thermal-induced Unfolding

DSC (Fig. 5) and CD (Fig. 3B–C) were used to follow the thermal-induced unfolding and the profiles were independent of both the temperature scan rate and the protein concentration (data not shown); however wild-type Sis1 and SYS aggregated when heated above 90°C at a concentration of 1.5 mg/mL (data not shown). Based on the data presented in Fig. 5A–B and Table 1, wild-type Sis1 and Sis1_Δ_124–174_ were classified together since they presented two overlapping transitions in the thermal-unfolding profile, with similar thermodynamic constants for both transitions (Tms and *ΔH* ^cal^ – see Table 1). Sis1_Δ_121–257_ and SYS, which are the proteins with more structural dissimilarities in comparison to wild-type Sis1 (see Fig. 1), showed two well-separated thermal-unfolding transitions and also some similarities in their thermodynamic constants (Table 1). *ΔCp* was calculated from the Kirchoff plot (Fig. 5E–F), which describes the dependence of *ΔH* ^cal^ with *Tm* in the presence of a denaturing agent. Fig. 5E–F shows the Kirchoff plot for all proteins in the first and second thermal-unfolding transitions, respectively, and their fits by Eq. 3 (Materials and methods) from which the values for *ΔCp*_1_ and *ΔCp*_2_ were calculated and are presented in Table 1.

During thermal-induced unfolding followed by CD, all proteins started to unfold at a temperature around 55°C, with Sis1_Δ_121–257_ being the sole exception, beginning to unfold at approximately 45°C. All proteins were completely unfolded at approximately 75°C (Fig. 3B–C). When [θ] was converted to delta of signal (Fig. 3C) we could evaluate the *Tm* of these thermal-induced unfolding transitions (*Tm_CD_*) by fitting using sigmoidal functions (Table 1). The *Tm_CD_*s for wild-type Sis1 and Sis1_Δ_124–174_ were similar to the *Tm_1_*s measured by DSC but the *Tm_CD_*s for Sis1_Δ_121–257_ and SYS were much higher than the *Tm_1_*s measured by DSC (Table 1).

### Fluorescence and NMR

A conclusion from the above results was that the G/M region did not contribute to the stability of Sis1 and thus further investigations of Sis1_Δ_124–174_ were made to yield more information on the contribution of this region to its conformation. Sis1 has a single tryptophan (Trp225) residue, which is located within the hydrophobic core of the CTDI (Fig. 1) and is still present in Sis1_Δ_124–174_ but not in the other mutants. To investigate whether the truncation caused conformational changes in the Trp225 environment a series of fluorescence studies were performed (Fig. 6). The emission fluorescence spectrum (Fig. 6A), anisotropy (Fig. 6B) and fluorescence quenching (Fig. 6C) for wild-type Sis1 and Sis1_Δ_124–174_ were recorded and compared showing that the Trp225 experienced the same environment in both the wt and mutant. The maximum emission wavelength and the center of spectral mass for wild-type Sis1 and Sis1_Δ_124–174_ (Table 2) were obtained yielding a value of 343 nm for both proteins, indicating a partially exposed tryptophan residue. As shown in Fig. 6B, the anisotropy values at various excitation wavelengths for the wild-type and mutant protein had no significant changes. Differences between the structural flexibility of wild-type Sis1 and Sis_Δ_124–174_ were investigated by dynamic processes, such as tryptophan fluorescence quenching (Fig. 6C) and both of their Stern-Volmer constant values were very similar (Table 2).

To gain information on the conformation of the G/M region, wild-type Sis1 and Sis1_Δ_124–174_ were labeled with ^2^H and ^15^N for TROSY NMR experiments. It was evident from the superposition of the TROSY NMR spectra that Sis1_Δ_124–174_ presented less signals than the wild-type due to deletion of the G/M region, and that most of the lost signals were in the central region of the TROSY spectrum with chemical shifts around 8.2 (Fig. 7). Moreover, most of the TROSY cross-peaks showing high chemical shift dispersion are superimposable between the two spectra. These data indicate that the deletion of the G/M region did not change the structure of the other domains, and that the GM region is intrinsically disordered (see Discussion).

## Discussion

### The Central Domains are Essential for the Chaperone Activity

In a previous work [22] we showed that Sis1_124–174_ and Sis1_121–257_ are as efficient as wild type Sis1 in stimulating the ATP hydrolysis of Ssa1, an expected result because the stimulatory action of J domain proteins on the ATPase activity of Hsp70/Ssa1 is substoichiometric, as previously shown [30], [31]. Thus the stimulation of the ATPase activity depends mainly on the presence of the J domain, which was intact in all the mutants studied in this and the previous work [22]. Here, using a direct assay, we show that Sis1_124–174_ was as efficient as wild type Sis1 in binding Ssa1 while Sis1_121–257_ was only partially efficient. However, we cannot overrule the hypothesis that steric constrains due to different domain compositions could be influencing the results.

Additionally, Sis1_124–174_ had about half of the ability of wild type Sis1 in refolding luciferase in the presence of Ssa1 while Sis1_121–257_ had no effect at all. These results are in good agreement with that of our previous work [22] showing that Sis1_124–174_ and Sis1_121–257_ are less efficient than wild type Sis1 in binding luciferase. These results suggest that the C-terminal domain plays a more dominant role in binding to Ssa1 than the GM domain, but that both domains are involved in directing protein substrates for refolding. Furthermore, the lower refolding efficiencies of the mutants could be a result of lower binding efficiencies, and therefore, delivery to Hsp70. These lower efficiencies could be explained by two possibilities. First, the absence of both the G/M region and the CTDI affects substrate selection, or secondly, the size and structure of Sis1_Δ_121–257_ is smaller than wild-type Sis1 [22] and this may impair its interaction with and/or the relative orientation to Hsp70 Ssa1 and consequently effect substrate transfer. Interestingly, the SYS chimera aided the refolding of the heat-denatured luciferase almost twice as robustly as the wild-type Sis1, but similarly to that observed in wild-type Ydj1 (this work and [19]). These results are in good agreement with those of the binding experiments: Sis1 and YSY had approximately 15% and 20%, respectively, less binding efficiency for unfolded luciferase than Ydj1, whereas SYS was approximately 10% more efficient in binding unfolded luciferase than Ydj1 [19]. Due to the relevance of the central region for DnaJ function and structure we therefore deemed it important to study the conformation and stability of the deletion and chimeric mutants in more detail.

### The G/M Region is Intrinsically Disordered, thus not Contributing to the Stability or to the Global Conformation of Sis1

Although Sis1_Δ_124–174_ differs from wild-type Sis1 by the absence of the G/M region, wild-type Sis1 and Sis1_Δ_124–174_ presented overlapping transitions in both the urea- and thermal-unfolding profile, with similar parameters (*Cm*s, *Tm*s and *ΔH* ^cal^). Biophysical experiments exploring the intrinsic fluorescence emission of the single tryptophan present in the CTDI of wild-type Sis1 and Sis1_Δ_124–174_ also showed that these proteins present similar properties, such as anisotropy and tryptophan exposure to solvent as well as to quenchers. The Stern-Volmer constants (*K_SV_*) for collisional quenching processes express the susceptibility of the fluorophore to the quencher agent and higher values are associated with more exposed or flexible fluorophores. The higher values of *K_SV_* at 40°C for both proteins validates our method of analysis, showing that quenching processes occur between the tryptophan residues of these proteins, and that the quenching process is collisional. The anisotropy values at various excitation wavelengths for the wild-type protein and the mutant protein had no significant changes, indicating that the absence of the G/M region does not affect the rotational diffusion behavior of these proteins. SAXS and SV-AUC data also show that wild-type Sis1 and Sis1_Δ_124–174_ were structurally similar [22]. In fact, every investigation that compared the global conformation and stability of wild-type Sis1 and Sis1_Δ_124–174_ indicated that the proteins had similar behavior (this work and [22]).

To confirm these data, and to gain insight into the secondary structure of the G/M domain, we performed TROSY NMR experiments with ^2^H ^15^N labeled wild-type Sis1 and Sis1_Δ_124–174_. The superposed TROSY NMR spectra of wild-type Sis1 and Sis1_Δ_124–174_ showed that most of the TROSY cross-peaks showing high chemical shift dispersion are superimposable between the two spectra, indicating that the deletion did not change the structure of the folded domains. The results presented suggest that the G/M region was an intrinsically disordered region and should be flexible. This is an interestingly result that corroborates with several other studies which have also shown flexibility in Sis1 [21], [22] and adds to other observations of intrinsically unfolded regions being involved in chaperone function. A possible model that combines these observations is shown in Fig. 8A suggesting that the disordered G/M region participates in the binding and delivery of client proteins to Hsp70 and thus is essential to Sis1 function. It is important to note that our TROSY NMR analysis was performed on substrate free Sis1, and that the G/M region may gain structure upon substrate binding.

### Sis1 Unfolds via a Partially Folded Monomer

The urea-induced unfolding of Sis1 was studied by two experiments (SEC-MALLS and sedimentation velocity AUC) which investigated the molecular mass of the protein. Since almost 50% of the secondary structure is lost in 4 M urea, a concentration in which the protein behaved as a monomer, we suggest that the dimer to monomer transition occurred concomitantly with partial unfolding (see below). Important considerations are raised from these analyses. First, the fact that Sis1_Δ_124–174_ behaved similarly to the wild-type was another indication that the G/M region is intrinsically disordered and did not contribute to the folded state or to the dimer form (see below). Second, since the C-terminus is involved in dimer formation [16], a possible hypothesis to describe the unfolding of Sis1 can be understood by considering that the unfolding of the C-terminal domains occurs first, forming a partially unfolded monomer in which the N-terminal domains are structured. These hypotheses are supported by several lines of evidence shown in this work, allowing us to propose a general unfolding pathway for *Sis1* that involves at least 2 events (Fig. 8B). The first unfolding event is the unfolding of the CTD that, consequently, leads to dimer dissociation. The first event forms an intermediate composed of partially folded monomers in which the J-domain still remains folded.

### Additional Contacts in the C-terminal Region Contribute to the Stability of the Sis1 Dimer

In agreement with our unfolding hypothesis, our DSC experiments clearly showed that at least two events were taking place during the thermal-induced unfolding of Sis1, although the two events are less well separated in the wild-type Sis1 and Sis1_Δ_124–174_ profiles than in the Sis1_Δ_121–257_ and SYS profiles, as the first event in the latter occurred at a much lower temperature. Nonetheless, our hypothesis is corroborated by several observations, summarized in Table 3. First, *ΔCp2* was similar for all proteins studied. Since *ΔCp* is related to the exposure of hydrophobic surfaces during thermal-induced unfolding, and similar structural domains should expose similar hydrophobic surfaces during unfolding, we conclude that *ΔCp2* was related to the unfolding of the J-domain and G/F region, while *ΔCp1* was considered to be related to the unfolding of dimeric Sis1 into partially unfolded monomers. Secondly, Sis1_Δ_124–174_ and Sis1_Δ_121–257_ presented similar *ΔCp1*_residue_ values, which were almost two times greater than the *ΔCp1*_residue_ values obtained for wild-type Sis1. This data suggests that the CTD of Sis1_Δ_124–174_ and Sis1_Δ_121–257_ is well folded as in the wild-type and that their unfolding leads to a similar exposure to solvent. Additionally, it is known that CTDI and CTDII of Sis1 present similar structural folds [16], therefore their unfolding should be similar, resulting in the similar *ΔCp1*_residue_. Furthermore, we show that wild-type Sis1 presented a smaller *ΔCp1*_residue_ than Sis1_Δ_124–174_ suggesting that wild-type Sis1 possesses more amino acid residues exposed to water. Wild-type Sis1 differs from Sis1_Δ_124–174_ only by the presence of the G/M region that is likely intrinsically unfolded as demonstrated by other experiments in this work, thereby supporting the hypothesis that the G/M region of Sis1 was intrinsically unfolded, as argued above. Lastly, *ΔCp2*_residue_ should be similar among the proteins, and indeed this is what we found.

Previous studies have shown that dimerization of Sis1 is important for binding and directing protein substrates for refolding, as abrogation of the dimerization site results in a protein defective in these activities [16]. Hence, it is important to note the differences in the unfolding profiles of Sis1_Δ_124–174_ and Sis1_Δ_121–257_, which differ by the absence of the CTDI, because this can elucidate important information about how the two CTDs interact to maintain dimerization. Both thermal- and chemical-induced unfolding indicates that Sis1_Δ_124–174_ is more stable than Sis1_Δ_121–257_. This result led us to conclude that the CTDI should be important for stabilizing CTDII, which is responsible for Sis1 dimerization and vice versa. To understand how this occurs, we analyzed the crystallographic structure of the CTD of Sis1 [16] and observed 6 saline bridges and 14 hydrophobic amino acids (which form a hydrophobic core) between the CTDI and CTDII interface (Fig. 9 and Table 4). These contacts probably increase wild-type Sis1 and Sis1_Δ_124–174_ stability in thermal and chemical-induced unfolding experiments. The absence of the CTDI in Sis1_Δ_121–257_ likely compromises the structure of the CTDII and impairs its dimerization stability, while the reduced stability observed in the SYS chimera likely results from disruption of the native contacts as a result of the swapping. Therefore, contacts located in the CTD’s interface of the protomer in the dimer are important for stabilizing the CTD of Sis1 in the dimeric structure. Interestingly, stimulation of ATPase activity does not seem to be dependent upon dimerization, as a mutant Sis1 monomer incapable of forming dimers is fully capable of stimulating Hsp70 Ssa1 ATPase activity, and our previous results show no impairment in this activity with the Sis1 mutants studied here [16].

In the light of our results, one can conclude that the differences in dimer dissociation stability could be due to another dimerization binding site in Sis1, which could lie within the CTDI of each protomer. This hypothesis could be supported by the results shown here. However, crystallographic studies of Sis1 [16] and SAXS data [21], [22] did not show, unequivocally, the existence of a second dimerization binding site.

Taking into account the results presented in this report, we can also suggest the reason for the requirement of two similar subdomains forming the CTD in the DnaJ type II subfamily. It is known that the CTDI holds the substrate binding site of Sis1 [12] and CTDII holds the dimerization site [16]. Two subdomains with similar structural folding elements might be needed to establish enhanced stability, in a synergistic way, by the contacts made at the interfaces of the subdomain. This enhanced stability would help to maintain the CTDI structure, thereby also maintaining proper Hsp70/Ssa1 and substrate interactions under stressed conditions.

### Conclusion

Sis1 is an important model for studying the function of the DnaJ type II subfamily. Structural and functional studies have shown that Sis1 contains a bent horseshoe structure [16], which may be important for interacting with Hsp70 and/or substrates [4]. We showed that the two Sis1 deletion mutants (Sis1_Δ_124–174_ and Sis1_Δ_121–257_) exhibit impaired functions in terms of chaperone activity and the ability to cooperate with Hsp70/Ssa1 to refold luciferase. Based on the results obtained we proposed a general unfolding pathway for Sis1 and its mutants, which involves at least two events. The first event includes unfolding of the CTD, which in turn leads to dimer dissociation and produces an intermediate formed by a folded J-domain and unfolded CTD. The second event involves unfolding of the J-domain. We also show that the G/M region is intrinsically disordered in the absence of substrate, and that the CTDI is important for Sis1 function.
